# Immunogenicity of ChAd63 + MVA ME-TRAP in Senegalese adults

**DOI:** 10.1186/1475-2875-13-S1-P93

**Published:** 2014-09-22

**Authors:** Victorine Mensah, Danny Wright, Katie Ewer, Nick Edwards, Magatte Ndiaye, Phillip Bejon, Nicola Viebig, Babacar Faye, Adrian Hill, Badara Cisse

**Affiliations:** 1 Jenner Institute, Oxford University, Oxford, UK; 2 University Cheikh Anta Diop, Dakar, Senegal; 3 The KEMRI-Wellcome Trust Research Programme, Kilifi, Kenya; 4 UniversitätsKlinikum Heidelberg, Heidelberg, Germany

## Background

While malaria transmission is falling in many areas due to increased use of bed-nets and anti-malarials, it remains an enormous burden [1]. The leading malaria vaccine candidate, RTS,S, results in often considerably less than 60% efficacy against clinical malaria [2] resulting in a need for other approaches that may enhance this partial protection. One such approach is the use of viral vectors in a heterologous prime-boost regime with the aim of inducing a T-cell response. Previous studies have shown high levels of immunogenicity by using a chimpanzee adenovirus (ChAd63) followed by an attenuated vaccinia virus (modified vaccinia Ankara (MVA)) to deliver the pre-erythrocytic antigen, multiple epitope string with thrombospondin-related adhesion protein (ME-TRAP) [3].

## Materials and methods

We undertook a phase IIb study to assess the efficacy and further evaluate the immunogenicity and safety of such a vaccination strategy involving 120 healthy adults in Dakar, Senegal. Volunteers received either ChAd63 followed by MVA, both containing ME-TRAP, or two shots of rabies vaccine. After the second immunization, all volunteers were given short-lasting antimalarials. The time taken to first malaria infection was measured by PCR and compared between the two groups. IFN-γ production by T-cells, seen as an integral component of liver stage protection, was measured by ELISpot at various points throughout the trial. Intracellular cytokine staining was used to determine the relative proportions of CD4^+^ and CD8^+^ T-cells secreting IL-2, IFN-γ and/or TNFα in response to peptide stimulation.

## Results

Peak immunogenicity was identified 7 days after boosting vaccination with MVA ME-TRAP, at a geometric mean of 1266 spots per million PBMC (95% CI 985-1504) compared with 87 spots per million (95% CI 57-117) among control vaccinees, *P* < 0.0001. While this vaccine strategy is aimed at enhancing the cellular response towards the liver stage, TRAP-specific IgG responses were also measured by ELISA and were modest in magnitude. Efficacy analysis using time to PCR positive is ongoing, however this vaccine approach has shown an acceptable safety profile and good, potent cellular immunogenicity in this semi-immune population.
